# Ideas from the Frontline: Improvement Opportunities in Federally Qualified Health Centers

**DOI:** 10.1007/s11606-023-08294-1

**Published:** 2023-07-17

**Authors:** Olivia S. Jung, Fahima Begum, Andrea Dorbu, Sara J. Singer, Patricia Satterstrom

**Affiliations:** 1 Department of Health Policy and Management, Fielding School of Public Health, University of California, Los Angeles (UCLA), Los Angeles, CA USA; 2 Laboratory of Innovation Science at Harvard, Harvard University, Cambridge, MA USA; 3 Healthcare Transformation Lab, Massachusetts General Hospital, Boston, MA USA; 4 Harvard College, Harvard University, Cambridge, MA USA; 5 School of Medicine and Graduate School of Business, Stanford University, Stanford, CA USA; 6 Wagner Graduate School of Public Service, New York University, New York, NY USA

**Keywords:** quality improvement, innovation, innovation contests, ideas, frontline workers, employees, federally qualified health centers, primary care

## Abstract

**Background:**

Engaging frontline clinicians and staff in quality improvement is a promising bottom-up approach to transforming primary care practices. This may be especially true in federally qualified health centers (FQHCs) and similar safety-net settings where large-scale, top-down transformation efforts are often associated with declining worker morale and increasing burnout. Innovation contests, which decentralize problem-solving, can be used to involve frontline workers in idea generation and selection.

**Objective:**

We aimed to describe the ideas that frontline clinicians and staff suggested via organizational innovation contests in a national sample of 54 FQHCs.

**Interventions:**

Innovation contests solicited ideas for improving care from all frontline workers—regardless of professional expertise, job title, and organizational tenure and excluding those in senior management—and offered opportunities to vote on ideas.

**Participants:**

A total of 1,417 frontline workers across all participating FQHCs generated 2,271 improvement opportunities.

**Approaches:**

We performed a content analysis and organized the ideas into codes (e.g., standardization, workplace perks, new service, staff relationships, community development) and categories (e.g., operations, employees, patients).

**Key Results:**

Ideas from frontline workers in participating FQHCs called attention to standardization (*n* = 386, 17%), staffing (*n* = 244, 11%), patient experience (*n* = 223, 10%), staff training (*n* = 145, 6%), workplace perks (*n* = 142, 6%), compensation (*n* = 101, 5%), new service (*n* = 92, 4%), management-staff relationships (*n* = 82, 4%), and others. Voting results suggested that staffing resources, standardization, and patient communication were key issues among workers.

**Conclusions:**

Innovation contests generated numerous ideas for improvement from the frontline. It is likely that the issues described in this study have become even more salient today, as the COVID-19 pandemic has had devastating impacts on work environments and health/social needs of patients living in low-resourced communities. Continued work is needed to promote learning and information exchange about opportunities to improve and transform practices between policymakers, managers, and providers and staff at the frontlines.

**Supplementary Information:**

The online version contains supplementary material available at 10.1007/s11606-023-08294-1.

## INTRODUCTION

Frontline clinicians and staff, who regularly and directly interface with patients and workflows and do not hold senior management positions, are well-positioned to generate and voice ideas for improvement and to participate in organizational problem-solving.^1^ Frontline engagement in such activities has been shown to improve quality of care and patient safety.^2,3^ Engaging frontline workers to identify improvement opportunities may be particularly fruitful in federally qualified health centers (FQHCs) and similar safety-net settings, as large-scale, top-down transformation initiatives are often associated with declining morale and increasing burnout among frontline workers in these settings.^4,5^ Thus, it is important to understand what frontline workers want to see changed to improve work environments and care delivery processes.

Innovation contests, which decentralize problem-solving and innovation, can be used to involve frontline workers in quality improvement and innovation.^1,6^ While contests are a well-established mechanism for problem-solving,^7^ using them in the context of innovating healthcare delivery is relatively novel. Contests broadcast a call for ideas and invite willing and able individuals to contribute solutions.^8,9^ By inviting workers to generate ideas and then to vote on submitted ideas, contests draw on workers’ creativity and initiative.^10^ Contests can increase worker morale, by offering transparency in idea generation and selection processes and enabling workers to play a proactive role in solving organizational problems that are vexing to them. Moreover, contests have the potential to improve the quality of care when organizations act on the challenges identified and voted on by those at the frontlines. Conducting innovation contests with frontline workers is similar to comprising FQHC governing boards with patients and community members to seek bottom-up input.

In this paper, we describe the ideas that surfaced via innovation contests implemented in a national sample of 54 FQHCs. More than 1,400 workers generated nearly 2,300 ideas over a 3-week period. The ideas reveal what frontline workers, who are closest to patients and care delivery, identified as opportunities for improving care delivery processes and the experience of care among providers, staff, and patients.

## METHOD

This study is part of a multi-method research project that examined the impact of innovation contests on frontline employees’ voice and innovative behaviors in FQHCs.

### Study Sample

Out of the 1,367 FQHCs funded by the Health Resources and Services Administration (HRSA) that were invited, 54 directors signed an agreement to participate in this research project. Table 1 compares the characteristics of participating and non-participating FQHCs. Participating FQHCs were evenly distributed among the four US geographic regions, with 14/54 (26%) in the Northeast, 11/54 (20%) in the Midwest, 16/54 (30%) in the South, and 13/54 (24%) in the West. At the time of the study, participating FQHCs had 236 employees on average, ranging from 19 to 3,035. Larger FQHCs with multiple sites were encouraged to divide their employee pool by role or site and conduct a contest for each pool. On average, health centers hosted 2.25 contests, with the number ranging from 1 to 7. More than 70% (39/54) had obtained patient-centered medical home (PCMH) certification and 37% (20/54) had received a Quality Improvement Award from HRSA in 2017. All study procedures were approved by the Institutional Review Board at Harvard University.

**Table 1:** Participating versus Non-Participating FQHCs

	Participating FQHCs		Non-participating FQHCs		
	*N*	Mean (SD)	*N*	Mean (SD)	*p*-value
Organizational characteristics					
Number of delivery sites	54	9.56 (10.1)	1313	8.03 (8.99)	0.22
Total number of patients	54	25,384 (33,889)	1308	20,649 (24,846)	0.18
Amount of grant expenditure	54	3,804,186 (2,581,633)	1308	3,450,324 (2,668,990)	0.34
PCMH accreditation, (*N* (%))	54	39 (72)	1279	891 (70)	0.69
Advanced use of EHR, (*N* (%))	54	38 (70)	1279	871 (68)	0.73
QI award, (*N* (%))	54	20 (37)	1279	407 (32)	0.42
Patient population characteristics					
% patients living in poverty	54	63.7 (17.7)	1308	65.1 (18.5)	0.60
% uninsured patients	54	22.4 (14.6)	1308	25.0 (18.1)	0.30
% Black patients	54	21.6 (25.5)	1304	22.7 (26.2)	0.76
% Hispanic patients	54	24.5 (23.6)	1307	28.1 (27.8)	0.34
% Asian patients	54	2.53 (3.82)	1302	4.00 (10.6)	0.31
% mental health treatment patients	53	9.36 (8.91)	1284	8.94 (10.4)	0.77
% SUD treatment patients	45	1.11 (1.94)	1078	1.39 (3.45)	0.59
Clinical quality measures (% achieved)					
Adult weight screening and follow-up	54	70.9 (19.7)	1308	68.0 (21.6)	0.33
Heart attack/stroke treatment	54	79.9 (9.96)	1303	80.1 (13.4)	0.92
Asthma treatment	54	86.5 (12.2)	1303	85.6 (13.1)	0.60
Colorectal cancer screening	54	39.6 (18.3)	1306	40.7 (17.4)	0.65
Cervical cancer screening	54	53.3 (154.1)	1307	50.9 (17.1)	0.29
Cholesterol treatment	54	81.8 (8.74)	1303	80.6 (12.3)	0.47
Blood pressure control	54	62.1 (9.64)	1308	62.5 (10.14)	0.76
Low birthweight delivery	50	8.99 (7.77)	1188	9.35 (11.5)	0.83
Access to prenatal care	52	73.8 (15.6)	1241	76.8 (16.3)	0.19
Childhood immunization	53	30.9 (20.1)	1263	34.3 (22.7)	0.28

*EHR* electronic health record (an advanced use of EHR refers to employing EHRs to report on all clinical quality measures for all of health center’s patients), *PCMH* patient-centered medical home, *QI* quality improvement (a QI award refers to being recognized by HRSA for achieving the best overall clinical performance among all FQHCs), *SUD* substance use disorder. A chi-square test was used to compare the PCMH accreditation, advanced use of EHR, and QI award. For all other variables, Student’s *t*-test was used

### Innovation Contests

The first author coordinated with a senior manager (e.g., chief executive officer, director of human resources) from each FQHC to implement the organizational innovation contests, which we called an “Ideation Challenge.” The contests broadly sought ideas to improve patient care. Participants competed for winner and runner-up prizes of $100 and $35 gift cards, respectively. To standardize the implementation of contests, the first author created the materials and language to promote and administer the contest and asked senior managers to use them at specified time points. Contests were promoted using emails, videos, and posters. In all promotional materials and language, we emphasized the ease of access and participation (e.g., comparing participation to posting on Twitter).

All employees, regardless of role and tenure in the organization and excluding those in senior management, were invited to participate. The contests took place virtually in late 2018 through early 2019. They began with a 3-week-long ideation phase. Ideation entailed visiting the contest website and the idea submission page, on one’s computer or smartphone, to describe a work issue and propose a solution, with each response limited to 250 characters. Participants were also asked to provide a short summary of their idea, to be showcased on the voting platform. Next came a 3-week-long voting phase. Participants were asked to visit the same website, read the idea summary, and indicate the extent to which they would like to see each idea implemented, on a scale of 1 (do not want to see it implemented) to 5 (want to see it implemented). The average of the ratings determined the winners. Figure A1 in the Supplementary Appendix shows screenshots of the contest platform.

Apart from employee voting, we asked senior managers to evaluate the submitted ideas in terms of novelty, feasibility, and impact, following previous research.^11,12^ Senior managers from 53 FQHCs evaluated the ideas from their own organization in these dimensions on a scale of 1 (low) and 5 (high), without knowing who submitted which idea or how the ideas fared in voting.

### Analysis

We performed a content analysis, which is used to organize large amounts of text into an efficient number of meaningful categories.^13,14^ We conducted two rounds of coding. In the first round, the first and second authors read through the submissions, including the problems that the participating employees identified, the solutions they generated to address those problems, as well as the summary of the ideas as displayed on the voting platform. Together, the two authors read through 539 problems and corresponding ideas and noted recurring topics, such as standardization, patient experience, compensation, and new service. In this way, the two authors inductively developed a list of codes and the definition of each code. This approach ensured that the knowledge generated from our analysis was grounded in the data, or participating FQHC employees’ unique perspectives. Then two authors split up the remaining submissions (the first author reviewed 872 and the second author reviewed 860) and continued to develop the list of codes while also deductively coding the submissions using the emerging coding scheme. The two authors met once a week to discuss their progress on coding, consider adding or modifying the codes, and refine their definitions. After going through all of the ideas, the two authors sorted codes with conceptual overlaps and similarities into categories. For example, standardization, office space and supply, scheduling and access, technology to aid operations, and quality metrics were categorized as “operations.”

In the second round of coding, the first and second authors split up the work of reviewing ideas by code. For example, the first author reviewed the ideas filed under staffing and staff training, while the second author reviewed the ideas filed under workplace perks and compensation. The two authors checked that the ideas were appropriately coded and organized, according to the coding scheme that emerged from the first round of coding. The two authors flagged ideas that needed to be recategorized and met weekly to determine whether to apply new codes or keep existing ones.

With data on employee voting and senior manager evaluation of ideas, we standardized the scores for each of the four dimensions—(1) want to see implemented, according to employees and (2) most novel, (3) most feasible, and (4) most impactful, according to senior managers—to facilitate comparisons across clinics. For each dimension, we computed the mean score and standard deviation for each clinic. We then subtracted the raw scores by the mean clinic score and divided this difference by the standard deviation.

## RESULTS

Contests generated 2,271 ideas from 1,417 frontline clinicians and staff across all participating FQHCs. On average, 15% of FQHC employees contributed ideas, with participation in ideation ranging from 2.7 to 40%. While 41% of employees, on average, participated in voting, this value ranged from 12 to 79% among FQHCs. These response rates are in line or higher than the response rates for similar innovation contests conducted in hospitals.^1^ Various role groups participated; of those who participated in idea submission and for whom we had information on role, 40% were non-clinical support staff (e.g., receptionists, referral coordinators, billing personnel), 22% were clinical support staff (e.g., medical assistants, dental assistants), 13% were health professionals (e.g., dietitians, behavioral health therapists), 9% were advanced practice nurses, 8% were physicians and dentists, and 6% were nurses.

**Figure 1:**
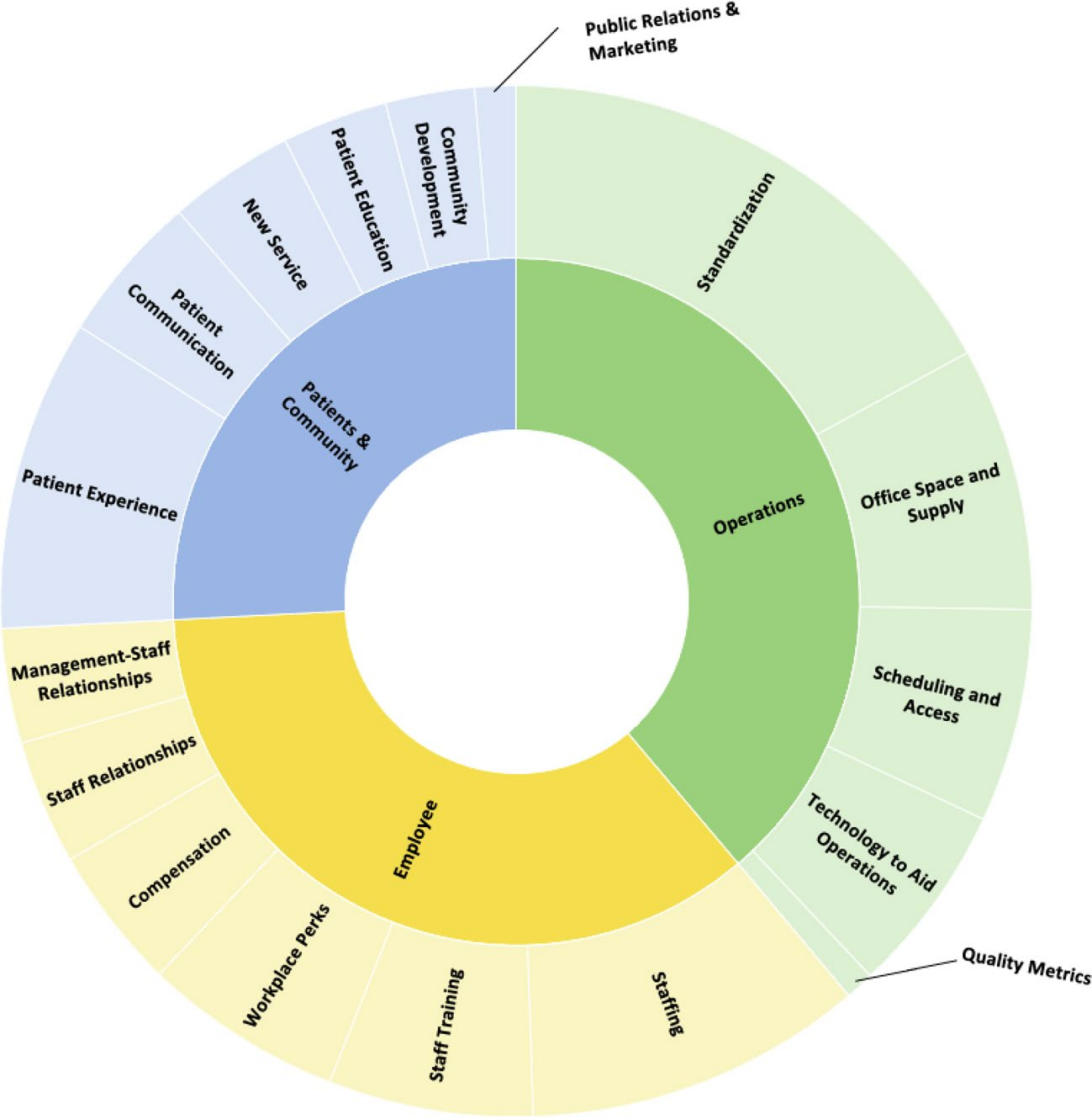
**Distribution of ideas in categories and codes.**

The ideas from frontline FQHC clinicians and staff called attention to the following thematic categories: operational improvement (*n* = 881, 39%), employees (*n* = 802, 35%), and patients and community (*n* = 588, 26%). Figure 1 displays the distribution of the codes and categories.

### Ideas on Operations

Table 2 presents the ideas calling attention to operational improvements, wherein ideas suggested improving the day-to-day functioning and activities of the health centers. Within this category, the most number of ideas pertained to standardization, with 386 (17%) ideas calling for standardizing and updating operational guidelines and evaluating existing workflows to create new protocols. Example ideas in this category included creating a “master index sheet of policies and approval/revision date that staff can quickly access to see if any policies have changed” and having a “checklist for registration as it is crucial to verify things like wrong birthday or spelling errors on patient names.”

**Table 2:** Ideas on Operations (*N* = 881, 39%)

Code	Ideas calling for	% (*N*)	Illustrative examples
Standardization	Standardizing and updating operational guidelines; evaluating and improving workflow	386 (17.0)	• Develop a standardized method for receiving/sending all medication refill requests at all locations • Make sure all policies are up to date, then hold training with management at each site • Choose a group of patient-facing staff to review/update forms used for registration
Office space and supply	Installing or replacing equipment, space for care delivery, effective inventory management	188 (8.3)	• Can we increase the thickness of the walls? Put in a sound barrier? Move the location of the counselors? We can hear our behavioral health patients’ private counseling sessions through the wall • Submit an email to all stating that you have an excess of this or that before it expires or before it is thrown away
Scheduling and access	Changing the hours of clinic operation and lengths of appointments to meet patient needs	151 (6.7)	• We live in a low income/cash pay only community. Visits need more time per patient to address more concerns • Allow flex scheduling (not just 8–5), which will help clients come in at times that do not interfere with their jobs
Technology to aid operations	New technology (e.g., electronic kiosks, tablets, upgrades to the electronic medical record to enhance operations	136 (6.0)	• Transform our clinics into a less wasteful and more energy efficient space by using tablets for paperwork • Create a kiosk or electronic check-in system for clients to address the busy front reception area and avoid any potential negative interactions with front desk staff • The current EMR system does not allow us to run real-time reports or print forms with one click. Consider a different system like Epic
Quality metrics	Improving measures on quality and other performance metrics	20 (0.9)	• Conduct monthly PDSA groups to study and initiate interventions to enhance outcomes, productivity requirements, and patient-centered care indicators • Create an organization-wide intranet that shows a dashboard of key performance indicators (patients per day/per site, financial indicators, patient satisfaction scores, etc.)

The second most-applied code in this category called for improved office space and supply (*n* = 188, 8%), by installing or replacing equipment, enhancing physical spaces for care delivery, and effectively managing inventory. Example ideas included “more or brighter lighting in the parking lot for visibility and safety” and “signs in the front office and/or exam rooms, in Spanish and English, that explain teen patients’ right to confidentiality for sensitive services.”

The third most-applied code called for improved scheduling and access (*n* = 151, 7%), by changing the hours of the clinic operation and lengths of appointments to meet patient needs. Example ideas included “4-day workweek: stay open Mon-Thurs for 10 h” and having “more available appointments by having a resident-run clinic.”

Next, ideas called for new technology and upgrading electronic medical records to enhance operations (*n* = 136, 6%). Example ideas included “enabling our website to drop registration information directly to NextGen so the information does not have to be entered manually” and “programming our new copier/fax machines to send faxes at the touch of a button!”.

Lastly, ideas called for improving measures on quality and other performance metrics (*n* = 20, 1%). An example idea was to “run reports of all patients treated at each clinic in the past two years and evaluate vaccine compliance. Send reminders to patients of the needed vaccines and wellness screenings, which are often free to patients.”

### Ideas on Employees

Table 3 presents the ideas pertaining to employees. The most-applied code in this category called for hiring more staff and designating staff to fill in certain tasks (*n* = 244, 11%). Example ideas included hiring “a social worker, preferably one who can speak Marshallese to be our liaison for the Marshallese population” and hiring “a psychiatric CRNP or PA for all sites, to enable completely integrated care on site.”

**Table 3:** Ideas on Employees (*N* = 802, 35%)

Code	Ideas calling for	% (*N*)	Illustrative examples
Staffing	Addressing staff shortage, hiring people, designating staff to fill in certain roles	244 (10.7)	• Hire more referral coordinators • Have additional staff at front desk to make the patient check-in process more efficient • A nurse practitioner who could float to multiple clinics one to 2 days a week to help with patient overload
Staff training	Providing training, encouraging staff to further education, creating professional growth opportunities	145 (6.4)	• Create a promotion path for entry/mid-level staff by offering training that expand skills and job scope • Offer classes, especially to non-clinical staff, to improve and obtain knowledge to grow within • Provide training on quality measures, as to why they are important and how they affect our operations
Workplace perks	Non-financial offerings to recognize hard work, increase morale, alleviate burnout, promote joy at work	142 (6.3)	• Employee of the month: staff nominate other staff who helped them or has gone above and beyond with patients. Winners get premier parking for the month • Clinics choose a day to celebrate each month (e.g., hat day, pizza party day, love people day). Decorate and dress up for that day • Celebrate social workers and case managers during the National Professional Social Work Month in March
Compensation	Increased wages, improved policies related to paid time off (PTO), educational support	101 (4.5)	• Giving staff not only a cost-of-living raise but a merit raise as well • Employees should have the option to be paid out for vacation time at the end of the year instead of losing it. When short-staffed, taking time off may not be an option • Offer 8 h per year for employees to use during extreme weather days
Staff relationships	Improving communication and building a sense of camaraderie among staff	88 (3.9)	• An employee obstacle course activity/puzzle/escape room to reduce division of departments • Keep an online social calendar for staff. Include events like local charity walks, volunteer opportunities, gathering at a dog park, etc. Aim for 1 event every 1–3 months
Management-staff relationships	Improving communication between managers and staff, increasing staff voice in organizational governance	82 (3.6)	• A “shadow day” between staff and managers with the goal of the manager seeing day-to-day work • Have someone from administration make rounds once or twice a quarter to discuss any concerns with frontline staff • I’d like to see a forum, where all can openly communicate about ideas and agency goals. I would like to see this for all levels, from the board, to c-level, to daily employees

The next most-applied code in this category pertained to staff training, with ideas calling for more training, reviewing staff competencies, encouraging staff to further their education, and creating professional growth opportunities (*n* = 145, 6%). Example ideas included developing “a MA ladder, similar to the LPN ladder, with opportunities to grow responsibility and increase pay after annual evaluations,” and “cross-training staff for front and back offices and billing for people to better understand how others do their job.”

The third most-applied code pertained to workplace perks, or non-wage/financial offerings that can recognize hard work, increase morale, alleviate burnout, and promote joy at work (n=142, 6%). Example ideas included “giving out stickers when an employee is observed doing something positive, e.g., help when not asked, show compassion” and providing “ergonomic supplies that improve posture, as many employees experience body aches and fatigue.”

The fourth most-applied code called for improved compensation (*n* = 101, 5%), in the form of increased wages and enhanced policies related to paid time off. Example ideas included instituting “bonus incentives for employees that not only meet their productivity expectation but surpass it” and “granting each full-time employee three sick days each year. We use vacation days when we are sick and often come into work sick.”

The next two categories of ideas called for cultivating staff relationships (*n* = 88, 4%) and management-staff relationships (*n* = 82, 4%). Cultivating staff relationships entailed improving communication and building a sense of camaraderie among employees. Example ideas included having “each clinic share about one individual (e.g., his or her favorite food, hobby) once a month so that we can get to know colleagues across clinics” and “coordinating family-friendly events like move nights or a day at a park. Staff don’t know each other very well and this can create barriers during the workday.” Cultivating management-staff relationships entailed improving communication between managers and staff and increasing staff voice in organizational governance. Example ideas included “having chiefs (e.g., CEO, CMO, COO) with one assistant take turns to make rounds, shadow, and observe clinics to get a real feel to make change” and “each site could have an anonymous suggestion box for employees to anonymously share ideas, concerns, frustrations; there is no safe outlet for expressing them without fear of consequences.”

### Ideas on Patients and Community

Table 4 presents the ideas pertaining to patients and community. The most applied code in this category called for improving patient experiences with care delivery and clinic amenities (*n* = 223, 10%). Example ideas included, “including transgender people in posters and pamphlets to create a welcoming environment” and “creating a ‘welcomeness’ metric to see how patients feel in our clinics and improve upon it.”

**Table 4:** Ideas on Patients and Community (*N* = 588, 26%)

Code	Ideas calling for	% (*N*)	Illustrative examples
Patient experience	Improving patient experiences with care, FQHC amenities	223 (9.8)	• A loyalty program for patients like they do at supermarkets and hotels • Host baby showers with giveaways for moms in their 8th month of pregnancy • A joint medical-dental well-child visit for each year of age to help kids connects with needed care
Patient communication	Improving patient communication related to appointment reminders, lab results, procedures	106 (4.7)	• Send out text messages to confirm appointments • Share lab results only via the patient portal or at appointment so that patients can ask any questions to their provider directly
New service	Implementing a new service that would serve groups of patients or treat a disease or condition that the clinic has not served or treated previously	92 (4.0)	• Create a mobile “food pharmacy” to distribute healthy foods to our diabetic population. Develop the program with our staff dieticians • Write a grant to receive rapid HIV testing and hire a case worker to help those that test positive • Improve pediatric obesity management by creating a multidisciplinary group visit with a medical provider and a health educator
Patient education	Providing information to patients about their health, health care, developing educational materials	75 (3.3)	• Curate adolescent-focused health content on our website, as adolescents are more likely to find inaccurate information • A health education board in rooms that clients can read while waiting for provider • Teach evidence-based self-care strategies to help patients manage stress and anxiety
Community development	Increasing FQHC interaction and engagement with the surrounding community	63 (2.8)	• Train seniors as Community Health Workers, who can help “coach” students to improve their behavioral health issues • Organize a Community Wellness Day in our parking lot. Showcase all our services and offer screenings
Public relations and marketing	Managing public impressions of the FQHC, raising awareness about the FQHC through advertising	29 (1.3)	• We should have billboards, more commercials, radio advertisement, etc. to draw in our patients • Our funders tend to be seniors who will likely retire from philanthropy soon. Host educational tours for young perspective donors interested in community health • Post short videos on our website that introduce our team, services, and location

The second most-applied code called for improving patient communication related to appointment reminders, lab results, procedures, and finances (*n* = 106, 5%). An example idea included “having and enforcing strict policy for late patients, which backs up the flow. Patients on time have to wait longer to see the provider, causing frustration for patients and staff.”

The third most-applied code alluded to implementing new services that would serve new groups of patients or treat a new disease or condition (*n* = 92, 4%). Example ideas included, “a special team of providers to take the medical bus once a month to severely underserved areas” and “a Community Care Paramedic program to prevent utilization of the ER.”

The fourth most-applied code pertained to patient education, or developing educational materials and providing information to patients about their health and health care (*n* = 75, 3%). Example ideas included, “filming a grocery shopping experience with patients and cook a heart-healthy or diabetic-friendly meal on the patients’ budget” and “having educational videos in the waiting area and exam rooms that provide simplified pathophysiology and explain the importance of nutritional modifications and medication adherence.”

The next two codes related to communities that FQHCs serve. One code pertained to community development, or increasing FQHC’s interaction and engagement with the surrounding community (*n* = 63, 3%). Example ideas included “starting a summer reading program for high school students who need community service hours to read to children in the waiting room” and “working with local artists to showcase community-generated art, telling patients’ narratives on the walls of shelter and school-based clinics.” The other code pertained to public relations and marketing, or managing public impressions of the FQHC and raising awareness about the FQHC through advertising (*n* = 29, 1%). An example idea was “increasing awareness about our optometry services by inviting people to like our Facebook page and share photos of themselves in broken or old glasses.”

### Which Idea Categories Most Resonated With Employees and Senior Managers?

Table 5 shows the distribution of top 100 ideas as voted by frontline employees and evaluated by senior managers across FQHCs by code. Of the top 100 ideas that received the highest scores from employees voting (in terms of which ideas employees want to see implemented), 15 mentioned staffing, 11 mentioned office space/supply, 11 mentioned patient communication, and 11 called for increased standardization. Ideas applied with these codes were also seen as most feasible to implement, but not novel, according to senior managers. Ideas related to a new service or serving a new group of patients were seen as most novel by senior managers (*n* = 14), but only a few ideas (*n* = 4) rose to the top in employee voting. Similarly, ideas related to patient experience were seen as novel (*n* = 15), feasible (*n* = 11), and impactful (*n* = 14) by senior managers, but just six ideas rose to the top in employee voting.

**Table 5:** Distribution of Top 100 Ideas as Voted by Frontline Employees and Evaluated by Senior Managers

	Want to see implemented (in employees’ view)^a^	Most novel (in senior managers’ view)	Most feasible (in senior managers’ view)	Most impactful (in senior managers’ view)
Operations				
Standardization	***11***	*9*	***24***	***13***
Office space and supply	***11***	*8*	*8*	*6*
Scheduling and access	**5**	**3**	**5**	**5**
Technology to aid operations	*6*	**5**	**2**	***12***
Quality metrics	**0**	**0**	**0**	**0**
Employees				
Staffing	***15***	*10*	***12***	*6*
Staff training	*6*	**4**	***12***	*10*
Workplace perks	*7*	*10*	**4**	**2**
Compensation	*6*	**2**	**2**	**5**
Staff relationships	**1**	*7*	**3**	**0**
Management-staff relationships	**5**	**4**	*7*	**2**
Patients and community				
Patient experience	*6*	***15***	***11***	***14***
Patient communication	***11***	**3**	**3**	*9*
New service	**4**	***14***	**1**	*8*
Patient education	**3**	**0**	**4**	**4**
Community development	**1**	**5**	**2**	**2**
Public relations and marketing	**2**	**1**	**0**	**2**

Codes with fewer than or equal to five top 100 ideas are in bold. Codes with six to ten top 100 ideas are shaded in italics. Codes with more than 11 top 100 ideas are shaded in bold italics

## DISCUSSION

This study identified nearly 2,300 ideas proposing to improve patient care offered by more than 1,400 frontline clinicians and staff in 54 FQHCs in innovation contests over a 3-week period. These ideas are likely to be impactful, as frontline clinicians and staff have empirical knowledge about which issues need to be fixed and how to fix them. Leveraging these ideas has the potential to help shape managerial and policy priorities and help decision-makers determine which improvement opportunities to prioritize.

Nearly 40% of the ideas called for improving day-to-day clinical and administrative operations. Of these ideas, 44% called for increasing standardization, highlighting a critical need, from the perspective of frontline workers, to develop and implement consistent, systematized protocols, workflows, and operational guidelines. Moreover, 24 of the 100 ideas deemed as most feasible to implement according to senior managers also called attention to standardization. Thus, interventions that standardize work and address concerns about inconsistent daily functioning and activities of clinics may be relatively easy to implement and achieve operational efficiency and enhance provider and staff experiences. Interventions that clarify roles and responsibilities, reduce duplication of tasks, and standardize day-to-day processes, such as scheduling and screening, have been shown to be associated with improved clinician satisfaction and reduced worker intention to leave.^15,16^

More than 35% of the ideas pertained to supporting employees. Underlying these ideas may be a call for help from workers. Addressing this call could alleviate burnout and declining job satisfaction. Of note, only about one in ten ideas in this category called for increased wages and financial compensation. Many more ideas (about 40% of ideas in this category) focused on improving morale in other ways, such as cultivating a sense of camaraderie and work relationships with peers, improving communication with managers, promoting frontline worker voice in organizational governance, and offering workplace perks. These ideas suggest that managerial practices, such as organizing team-building exercises,^17^ creating outlets for employees to voice ideas to one another and to managers,^18,19^ and empowering them to participate in quality and organizational improvement projects^1,20^ may help employees to feel heard and supported, and also help organizations to develop and retain workers.

Nearly half of the ideas in the category of supporting employees pertained to staffing resources and training. It is unsurprising that frontline employees are concerned about recruiting, retaining, and training personnel, as provider and staff shortages and frequent turnover have been enduring critical challenges faced by FQHCs.^21–23^ Prior research suggests that workers in fully staffed clinics experience lower burnout and spend less time on work that someone with less training could do.^24^ Thus, policies that enhance staffing in safety-net clinics, such as financial incentives that support providers who choose to work in underserved communities, loan repayment programs, and greater visibility of FQHCs during training,^21,22^ may serve to help employees feel supported, in addition to assuaging frontline concerns about staff resources and building clinics’ capacity to provide quality and accessible care.

There were overlaps as well as discrepancies, in terms of the ideas that senior managers deemed to be most novel and impactful, and the ideas that frontline employees wanted to see implemented. For example, while managers found ideas that proposed new services to be most novel, employees did not. Instead, employees expressed desire for implementation of ideas pertaining to staffing, standardization, and office space and supply. This finding suggests that ideas generated from innovation contests may not all be exceedingly novel or groundbreaking, from the perspective of senior managers and contest administrators. Rather, many are likely to suggest incremental changes. Even so, these ideas suggest important improvements that enable clinicians and staff to work more effectively and efficiently, so that they can focus on delivering care.

Moreover, few ideas called for improving quality metrics, which are often the focus of some of the more visible and prominent initiatives, such as achieving a medical-home recognition^4,5^ or adopting advanced health information technology^25^, that FQHCs conduct. In fact, such initiatives have been shown to negatively impact frontline worker morale and burnout.^4,5^ The issues that frontline workers care about—operational improvements, adequate staffing, alleviating burnout—may need to be resolved first, or considered in tandem, to support frontline workers as they are the ones who carry out quality improvement work and implementation of large-scale practice transformation initiatives. Increasing federal funding or instituting additional quality improvement awards to tackle worker-identified issues may also be key to facilitating a range of improvements in FQHCs.

This study was conducted before the COVID-19 pandemic. It is likely that the issues discussed in this study have been exacerbated, as the pandemic has had devastating impacts on clinical work environments and on health and social needs of patients living in low-resource communities.^26,27^ Healthcare organizations and policymakers should continue to solicit improvement opportunities from frontline workers to learn from and connect with those who are closest to patients and day-to-day work, especially as clinical work contexts continue to evolve and grow in complexity.

These findings should be considered in light of the following limitations. Our sample size is small and there may be concerns that this sample of FQHCs, where the senior managers elected to conduct innovation contests, is not representative of a typical FQHC. However, we confirmed that FQHCs in our analytic sample are not different from non-participating ones in terms of various organizational characteristics, and patients’ sociodemographic characteristics, as well as performance in numerous quality metrics (Table 1). In addition, ideas are impactful only if they are implemented. Implementing ideas was not part of innovation contests. While it was not clear to what extent participating FQHCs had the capacity to implement ideas, organizations may need to allocate resources for implementation to fully realize the benefits of soliciting ideas from the frontline. Failure to support ideas reach implementation (or respond appropriately to ideas that cannot be implemented) may negatively impact how frontline workers respond to calls for ideas and innovation in the future.

There is desire nationally among policymakers, practitioners, and scholars to transform primary care practice and improve the quality of care in safety-net settings and beyond.^28^ This study shows that using innovation contests, frontline workers can voice ideas that can facilitate organizational learning and inform implementation of large-scale transformation efforts, which have often had negative impacts on morale and burnout among frontline workers. Continued work is needed to promote learning and information exchange about opportunities to improve and transform practices between policymakers, managers, and providers and staff at the frontlines.

### Supplementary Information

Below is the link to the electronic supplementary material.

Supplementary file1 (DOCX 803 kb)

## Data Availability

The datasets analyzed in the current study are available from the corresponding author on reasonable request.

## References

[1] Jung OS, Jackson J, Majmudar M, McCree P, Isselbacher EM (2022). Engaging frontline employees using innovation contests: Lessons from Massachusetts General Hospital. Healthcare..

[2] Jung OS, Aiken LH, Sloane DM (2023). Nurse Work Environment and Hospital-Onset Clostridioides difficile Infection. Med Care..

[3] Tucker AL, Singer SJ (2015). The Effectiveness of Management-By-Walking-Around: A Randomized Field Study. Prod Oper Manag..

[4] Friedberg MW, Reid RO, Timbie JW (2017). Federally Qualified Health Center Clinicians And Staff Increasingly Dissatisfied With Workplace Conditions. Health Aff..

[5] Nocon RS, Fairchild PC, Gao Y (2019). Provider and Staff Morale, Job Satisfaction, and Burnout over a 4-Year Medical Home Intervention. J Gen Intern Med..

[6] Malhotra A, Majchrzak A, Kesebi L, Looram S (2017). Developing Innovative Solutions Through Internal Crowdsourcing. MIT Sloan Manag Rev..

[7] Morgan J, Wang R (2010). Tournaments for ideas. Calif Manag Rev..

[8] Guinan E, Boudreau KJ, Lakhani KR (2013). Experiments in Open Innovation at Harvard Medical School. MIT Sloan Manag Rev..

[9] Jeppesen LB, Lakhani KR (2010). Marginality and Problem-Solving Effectiveness in Broadcast Search. Organ Sci..

[10] Jung OS, Blasco A, Lakhani KR (2020). Innovation contest: Effect of perceived support for learning on participation. Health Care Manag Rev..

[11] Poetz MK, Schreier M (2012). The Value of Crowdsourcing: Can Users Really Compete with Professionals in Generating New Product Ideas?. J Prod Innov Manag..

[12] Girotra K, Terwiesch C, Ulrich KT (2010). Idea Generation and the Quality of the Best Idea. Manag Sci..

[13] **Weber RP.** Basic Content Analysis. Thousand Oaks, CA: Sage Publications; 1990.

[14] Hsieh HF, Shannon SE (2005). Three Approaches to Qualitative Content Analysis. Qual Health Res..

[15] **Linzer M, Poplau S, Grossman E, et al.** A cluster randomized trial of interventions to improve work conditions and clinician burnout in primary care: results from the Healthy Work Place (HWP) study. J Gen Intern Med. 2015;30(8). 10.1007/s11606-015-3235-4 PMC4510236 25724571

[16] Thies K, Schiessl A, Khalid N, Hess AM, Harding K, Ward D (2020). Evaluation of a learning collaborative to advance team-based care in Federally Qualified Health Centers. BMJ Open Qual..

[17] Havyer RDA, Wingo MT, Comfere NI (2014). Teamwork Assessment in Internal Medicine: A Systematic Review of Validity Evidence and Outcomes. J Gen Intern Med..

[18] Satterstrom P, Kerrissey M, DiBenigno J (2021). The Voice Cultivation Process: How Team Members Can Help Upward Voice Live on to Implementation. Admin Sci Q..

[19] Kerrissey MJ, Hayirli TC, Bhanja A, Stark N, Hardy J, Peabody CR (2022). How psychological safety and feeling heard relate to burnout and adaptation amid uncertainty. Health Care Manag Rev..

[20] Jung OS, Graetz I, Dorner SC, Hayden EM (2023). Implementing a COVID-19 Virtual Observation Unit in Emergency Medicine: Frontline Clinician and Staff Experiences. Med Care Res Rev..

[21] Rosenblatt RA, Andrilla HC, Curtin T, Hart GL (2006). Shortages of Medical Personnel at Community Health Centers: Implications for Planned Expansion. JAMA..

[22] Han X, Ku L (2019). Enhancing Staffing In Rural Community Health Centers Can Help Improve Behavioral Health Care. Health Aff..

[23] **Lewis C, Getachew Y, Abrams MK, Doty MM.** Changes at Community Health Centers, and How Patients Are Benefiting: Results from the Commonwealth Fund National Survey of Federally Qualified Health Centers, 2013–2018. The Commonwealth Fund; 2019.

[24] Helfrich CD, Dolan ED, Simonetti J (2014). Elements of Team-Based Care in a Patient-Centered Medical Home Are Associated with Lower Burnout Among VA Primary Care Employees. J Gen Intern Med..

[25] Frimpong JA, Jackson BE, Stewart LM, Singh KP, Rivers PA, Bae S (2013). Health information technology capacity at federally qualified health centers: a mechanism for improving quality of care. BMC Health Serv Res..

[26] Lopez L, Hart LH, Katz MH (2021). Racial and Ethnic Health Disparities Related to COVID-19. JAMA..

[27] Morgantini LA, Naha U, Wang H (2020). Factors contributing to healthcare professional burnout during the COVID-19 pandemic: A rapid turnaround global survey. PLoS ONE..

[28] National Academies of Sciences (2021). Engineering, and Medicine.

