# Supplementary figures and images for: Ideas from the Frontline: Improvement Opportunities in Federally Qualified Health Centers
Source: J Gen Intern Med. 2023 Jul 17;38(13):2888–97. doi: 10.1007/s11606-023-08294-1 (PMC10593646; doi:10.1007/s11606-023-08294-1)

# SUPPLEMENTARY APPENDIX

## Figure A1. Screenshots of the Innovation Contest Platform


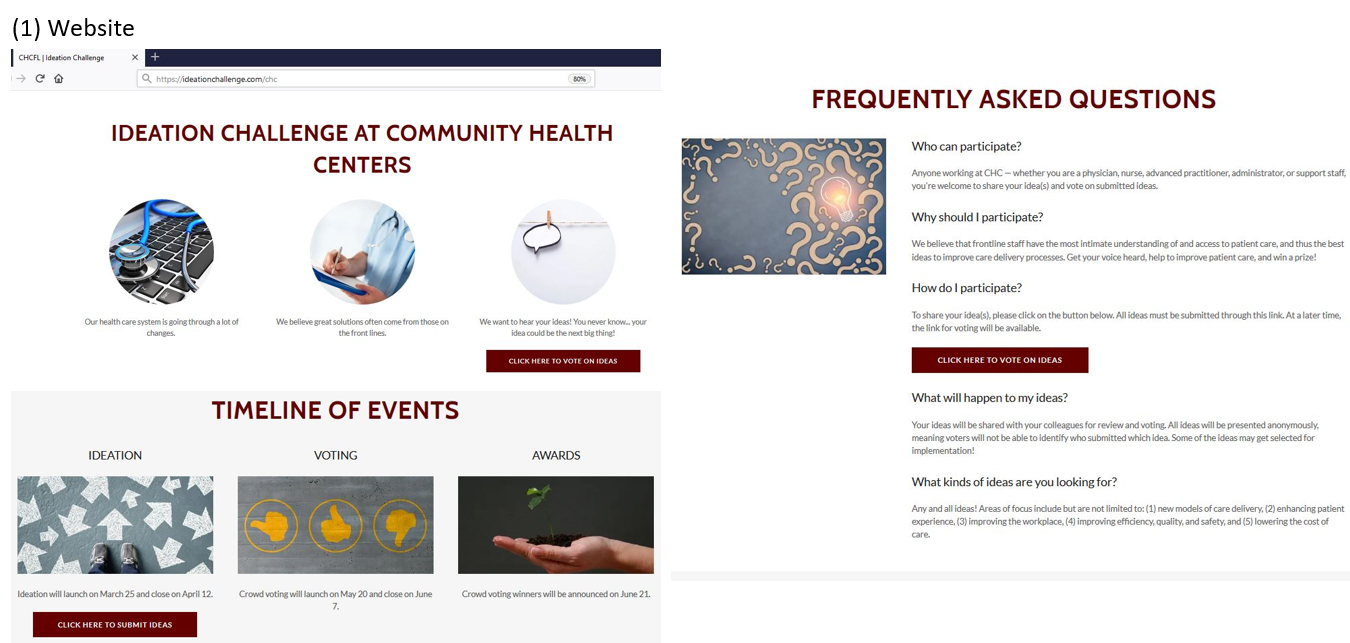


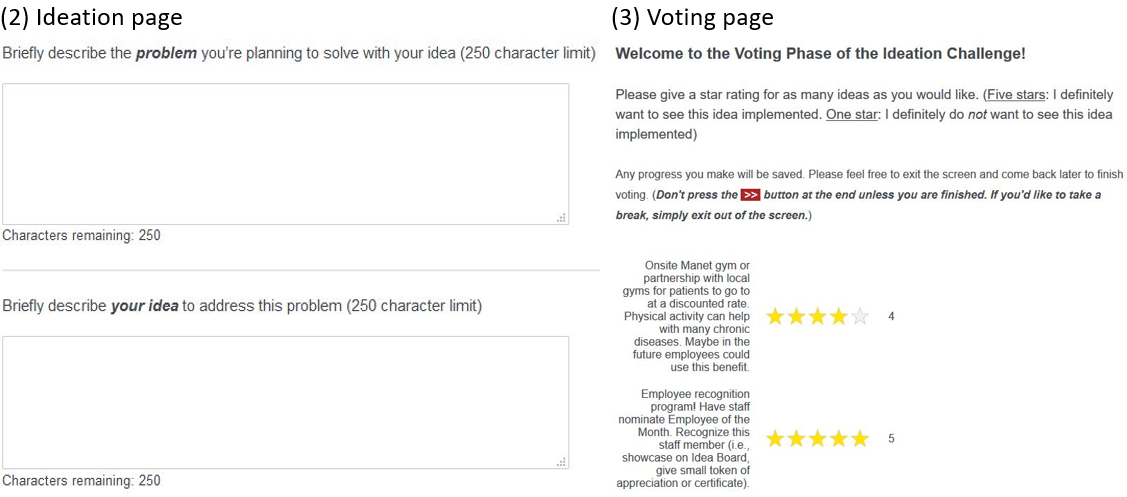

Supplement: Supplementary file 1 — Supplementary file1 (DOCX 803 kb) [file 11606_2023_8294_MOESM1_ESM.docx]
